# Money, Food, and Daily Life Objects Are Similarly Shared in the Dictator Game. A Study among Poles and Tsimane’

**DOI:** 10.3389/fpsyg.2017.00554

**Published:** 2017-04-12

**Authors:** Piotr Sorokowski, Anna Oleszkiewicz, Agnieszka Niemczyk, Michalina Marczak, Tomas Huanca, Esther C. Velasco, Agnieszka Sorokowska

**Affiliations:** ^1^Institute of Psychology, University of WrocŁawWrocŁaw, Poland; ^2^Smell and Taste Clinic, Department of Otorhinolaryngology, TU DresdenDresden, Germany; ^3^Centro Boliviano de Investigación y Desarrollo Socio IntegralSan Borja, Bolivia

**Keywords:** dictator game, money, food, sharing, generosity

## Abstract

The dictator game (DG) is one of the most popular methods for measuring sharing behaviors. However, the matter of goods used in the game has rarely been examined and discussed. We conducted a study in which all participants played standard version of DG in one of the three versions – “money,” “food,” or “daily life objects” sharing. Further, we wanted to expand the generalizability of our findings by investigating whether patterns in sharing various goods are independent of culture and the level of market integration. Thus, the study was conducted among people who function daily under the conditions of low market integration (109 Tsimane’ – forager-horticulturists from Bolivian Amazon) and in a society highly integrated with the market-based economy (85 Polish people). We observed that among both Polish and Tsimane’ people the participants were equally likely to share money, food and small, daily life objects with an unknown partner, which implies that generosity might not be related with the type of possessed resources. However, regardless of the kind of goods given, Tsimane’ people were less eager to share with anonymous others than Polish people. We present several implications of our findings for studies on generosity and altruism.

## Introduction

The dictator game (DG) is one of the most popular methods for measuring sharing behaviors (Engel, 2011). Many variations of the DG have been developed in order to capture different types of decisions and economic strategies. Originally developed by Kahneman et al. (1986), DG became very popular over the past 30 years, mostly because of its simplicity and accuracy in turning assumptions into measurable decisions (Engel, 2011). Based on observation of decisions made in this economic game, scientists came to the conclusion that people are more eager to share than *homo economicus* theory would suggest, i.e., the frequency and quantity of shared goods often exceeds the assumed, rational and self-centered social exchange (Fehr and Schmidt, 1999). To date, studies have shown that this pattern is observable across different cultures (Henrich et al., 2001).

Only by year 2009, DG was described in over 130 empirical papers, presenting over 616 various procedures and versions of the game (Engel, 2011). Differences included conditions of reciprocity (Ben-Ner et al., 2004b; Diekmann, 2004), degree of uncertainty and social distance between the players (Charness and Gneezy, 2008), partner’s gender and personality (Ben-Ner et al., 2004a), and minimal social cues (Rigdon et al., 2009). Nevertheless, the matter of goods used in the game has rarely been examined and discussed. In some studies, researchers used objects different than money (for example tobacco) to examine sharing patterns (e.g., Henrich et al., 2001), but possible effects and implications of this fact were not controlled. It seems quite surprising that to date this fundamental aspect of widely recognized measure of economic behaviors has not received enough scientific attention. Possibly, different goods of similar value used in canonical setting of DG can influence decisions of a player.

Former studies suggest that generosity might depend on monetary and non-monetary contexts. For example, it has been shown peoples’ inclinations to act pro-socially may be weaker in the contexts involving money (Vohs et al., 2006, 2008; Pfeffer and DeVoe, 2009). Relatedly, people seem to be more generous when involved in non-monetary exchange – for instance, they return the favor of a small gifts more often (Kube et al., 2012). Food exchange is also an important component of human cooperation and altruistic behavior (Kaplan et al., 1985, 2005). It developed earlier than money exchange in human history and in certain circumstances it is more often practiced. For example, some anthropologists argue that among Inuit hunter-gatherers living in the Canadian Arctic, food is exchanged more often than other goods or services (Kishigami, 2004).

In the light of above assumptions, it seems possible that the type of goods transferred within the DG might influence the willingness to share and that earlier studies involving DG could bring different results, if goods different than money were used (e.g., food or daily life objects). Therefore, we expected to observe a larger offer with non-monetary goods (or, more specifically, with foods). To test this prediction we conducted a study aimed at verification of the hypothesis that different types of goods involved in the DG can result in varied decisions on how much to share with a partner. Further, previous studies on DG were often conducted among participants from different cultures (Henrich et al., 2001; Gurven, 2004). Thus, we wanted to expand the generalizability of our findings by investigating whether patterns in sharing various goods are culturally independent.

## Materials and Methods

### Participants

To test the cross-cultural pattern of economic behaviors performed in DG with different types of goods involved, we collected data among people who function daily under the conditions of low market integration (Tsimane’ forager-horticulturists – Amazonian Indians) and in a society highly integrated with the market-based economy (Poland). Tsimane’ are a forager-horticulturists population of 8000 people scattered around a 100 villages in the region of Maniqui River (Huanca, 2006). Their level of integration with Bolivian economy, culture and society varies from a fairly traditional way of living (high isolation, performing shifting cultivation, hunting, fishing, and plant foraging) to relative integration (i.e., formal education, inhabiting settlements that can be reached by road, being regularly employed and relying on wages) (Godoy et al., 2010; Godoy, 2012). Poland is a country in Central Europe with a GDP of about 15,000 US$ per capita in 2015 (World Bank, 2016) and is a part of the European Union. It represents a modern industrialized society highly integrated with market economy.

The study was conducted among 109 Tsimane’, of whom 59 were females aged between 15 and 40 (*M* = 26.2, *SD* = 6.8) and 50 were males aged between 18 and 40 (*M* = 28.3, *SD* = 6.5). Tsimane’ participants were recruited among people living in four villages along the Maniqui River – Campo Bello, Las Palmas, Uasichi and Las Minas. These villages are located in similar proximity to the nearest town (approximately 3 h by canoe). Their inhabitants rely on foraging and small-scale agriculture, and only occasionally visit the town in order to sell surplus food products and purchase goods such as sugar and oil. We observed no significant differences in the level of market integration of the participants. The Polish sample comprised 85 participants of whom 45 were females aged between 17 and 39 (*M* = 21.6, *SD* = 3.7) and 40 were males aged between 18 and 30 (*M* = 21.7, *SD* = 2.6). Participants in Poland were recruited at university campuses in Wroclaw and Warsaw.

### Procedure

All participants played standard version of DG (Kahneman et al., 1986) in an experimental room (hut in case of Tsimane’) where their anonymity was secured. They were instructed that they were matched at random with another participant, and that they can share some money or goods with him or her. They were asked to decide on how much money or how many objects they want to give the partner and told that they can keep the remaining money or objects for themselves. Participants were randomly assigned to one of the three conditions – “money,” “food,” or “daily life object” (Tsimane: money – *n* = 38, food – *n* = 36, daily life object – *n* = 35; Poles: money – *n* = 29, food – *n* = 27, daily life object – *n* = 29); in each condition they could share 10 items. We instructed our participants that the study is anonymous, and that the recipient will not know who shared money, food or objects with them.

Polish participants in the first condition received 10 PLN (in 1 PLN coins) and were instructed to share it between anonymous partner and themselves. In the second condition the participants received 10 candy bars and in the third condition they were asked to share items useful in daily life (i.e., 10 pens). The value of all three types of items was approximately the same. Similar procedure was applied to Tsimane’, except that in this case the “money” were 10 bolivianos (BOB; in 1 boliviano coins), “food” items were 10 small packs of cookies, and “objects useful in daily life” were 10 fish hooks. Again, the value of all types of items was approximately the same. It is hard to compare if subjective values of our objects were smaller/higher for Tsimane or Poles. However, we would like to highlight that main aim of our study was not to compare results from this two populations.

### Ethics Statement

The study was conducted in accordance with the Declaration of Helsinki. The study protocol was approved by the Institutional Review Board (IRB) of the University of Wroclaw (Wroclaw, Poland) and by the Great Tsimane’ Council (the governing body of the Tsimane’). Polish participants provided written, informed consent prior to study inclusion, and due to the low levels of literacy among Tsimane’, we only obtained informed oral consent from the participants in this group.

## Results

All analyses were computed with IBM SPSS Software, version 22. Significant results of Shapiro-Wilk’s tests in both Tsimane’ and Polish samples across all three conditions indicated that the number of items transferred to the partner was not distributed normally (*p* < 0.001). Hence, in the further analyses we used non-parametric tests. For Mann–Whitney *U-*test we computed Hodges–Lehman estimation to obtain 95% confidence intervals. Significance level was set to alpha = 0.025, as we predicted higher generosity when sharing non-monetary goods.

In order to test the differences in generosity with different goods involved in the Polish sample we performed Kruskal–Wallis test with condition (“money,” “food,” or “daily life object”) as an independent factor and amount of money/quantity of objects given to the partner as a dependent variable. We found no differences between the conditions, *H*_2_ = 1.7, *p* = 0.43. Pairwise comparisons based on Mann–Whitney *U*-test indicate, that the effect sizes for each pair of conditions were marginal (food vs. money: *U* = 384.5, *p* = 0.9, η^2^ = 0.003; food vs. small object: *U* = 336.5, *p* = 0.34, η^2^ = 0.016; money vs. small object: *U* = 348, *p* = 0.21, η^2^ = 0.026; none of the pairwise comparisons survived Bonferroni correction). We also checked gender-related differences in generosity, but found no significant difference between men and women, *U* = 812, *p* = 0.4, η^2^ = 0.009, 95% CI [0.0, 0.0].

Analogous Kruskal–Wallis test in Tsimane’ sample revealed no differences in terms of shared goods quantity, *H*_2_ = 0.22, *p* = 0.90. Pairwise comparisons based on Mann–Whitney *U*-test indicate, that the effect sizes for each pair of conditions were again marginal (food vs. money: *U* = 657.5, *p* = 0.69, η^2^ = 0.003; food vs. small object: *U* = 629, *p* = 0.99, η^2^ = 0.0003; money vs. small object: *U* = 637.5, *p* = 0.68, η^2^ = 0.002; none of the comparisons survived Bonferroni correction). Interestingly, we observed a significant difference between women (*M*_rank_ = 60.8) and men (*M*_rank_ = 50.1), indicating lower generosity of the latter, *U* = 1183, *p* = 0.016, η^2^ = 0.05, 95% CI [0.0, 0.0].

**FIGURE 1:**
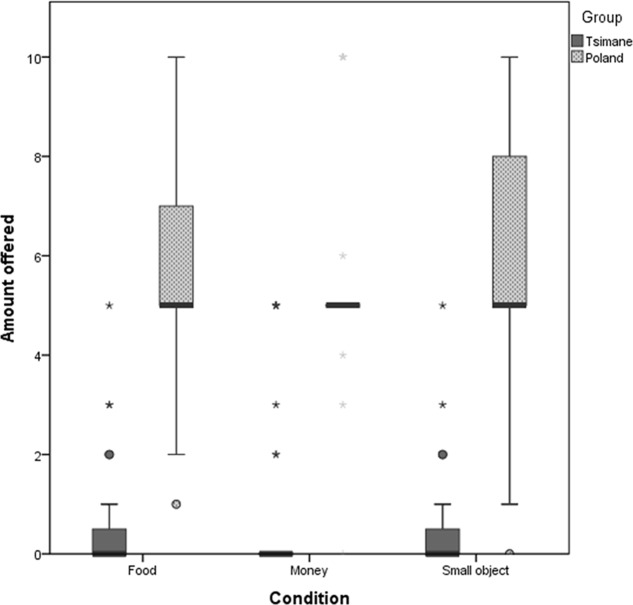
**Average amount of goods given to an anonymous partner in different versions of dictator game played by Tsimane’ and Poles.** Single outliers are marked with stars; error bars denote standard error values.

Finally, we compared Tsimane’ and Polish samples within each of the three conditions. We found significant differences in (a) “food” condition (*U* = 943.5, *p* < 0.001, η^2^ = 0.71, 95% CI [-5.0, -5.0]), showing lower tendency to share food in Tsimane’ (*Median* = 0) as compared to Poles (*Median* = 5); (b) “money” condition (*U* = 1029, *p* < 0.001, η^2^ = 0.65, 95% CI [-5.0, -5.0]) indicating lower tendency to share money with others in Tsimane’ (*Median* = 0) as compared to Poles (*Median* = 5); and (c) “daily life object” condition (*U* = 957, *p* < 0.001, η^2^ = 0.63, 95% CI [-7.0, -5.0]), showing lower tendency to share daily life objects in Tsimane’ (*Median* = 0) as compared to Poles (*Median* = 5). For mean values see: **Figure 1**.

## Discussion

Results of the current study indicate that applying different types of goods in the DG returned similar results among the Polish and Tsimane’ people. We observed that in both cultures, the participants were equally likely to share money, food and small, daily life objects with an unknown partner. The findings of our research are important for several reasons. First, this study suggests that generosity might not be related with the type of possessed resources, and second, it seems that type of applied goods in DG does not influence the level of generosity within cultures. Based on our research we can suggest that goods of similar objective value represent also similar subjective value to the participants, and that experimental DG paradigms can be created based on both monetary and non-monetary reward. Finally, our findings allow researchers to compare former results obtained with different types of goods of similar value.

The fact that in our study generosity did not depend on the type of shared resources seems to be rather surprising, because food sharing seems to be an especially important component of human cooperation and altruistic behavior (Kaplan et al., 1985, 2005; Bailey, 1991). Additionally, money may decrease the level of human pro-social orientation (Pfeffer and DeVoe, 2009; Gasiorowska and Helka, 2012) and can increase one’s efforts to attain personal goals (Vohs et al., 2006, 2008). Further, monetary and non-monetary reward often represent different values to the participants, most importantly because money can be exchanged for anything a person needs. Perhaps, the results we observed in our study resulted from all goods representing similar objective value to the participants, as (a) the amount of money that was to be shared was rather small, and (b) the non-monetary goods were quite useful to the participants. Perhaps, this equalized the subjective value of items applied in our experiment and led to similar outcomes across conditions. In future studies it can be investigated whether the objective value of applied items is actually reflected in subjective perception of the shared goods’ values.

Crucially, we found similar pattern of results across two culturally different samples of Poles and Tsimane’ – within each group, participants were equally likely to share each type of the possessed goods/items. The results create a space for the hypothesis, that the type of goods involved in the DG does not influence the level of generosity among players representing various cultures. To test such hypothesis, further studies involving participants representing more diverse cultures (both traditional and western) should be conducted.

It needs to be noted that regardless of the kind of goods given, Tsimane’ people were less eager to share with anonymous others than Polish people. These results remain in line with the former findings showing that the degree of market integration together with the payoffs to cooperation are positively correlated with the level of observed cooperation in experimental economic games (Henrich et al., 2001). It is also possible, that the goods provided by the experimenter represented higher subjective value to the Tsimane’ participants than to the Polish participants, and this is why the former were less likely to share the items with an unknown person. On the other hand, metaanalyses suggest that in traditional societies, dictators are significantly more generous as compared to players from Western, highly developed countries (Engel, 2011). However, these sources are based on a limited number of studies on economic behaviors conducted among members of primal societies, and the knowledge on patterns of their economic decisions remains rather scarce. Further investigations are required to fully understand cultural foundations on generosity presented in monetary and non-monetary contexts.

Interestingly, we found that in Tsimane’, men were less eager to share than women. This is rather an expected result (Engel, 2011) that remains in line with former findings suggesting, that women are typically less selfish than men (Eckel and Grossman, 1998). This difference might result from women being more oriented toward others and concentrated on interpersonal relations as compared to men, who are focused more on their own competence and goal achievements (Eagly, 2009). As majority of studies conducted in Western countries suggested that in women are more generous in DG than men (Engel, 2011) our result among Poland should be perceived as rare exception.

Finally, we observed extremely low readiness to share among Tsimane’. In the previous study conducted among Tsimane’ by Gurven (2004) the mean offer given in the DG was 32%, while here it was 5.9% (average for all types of goods declared to share). Similar to the study conducted by Gurven (2004), in our study economic games played among Tsimane’ were one-shot decisions performed under anonymous conditions, which should therefore eliminate any motivation to share based on status or reputation of the potential partner. We did not involve reciprocity setting, that could raise more altruistic decisions based on anticipated return from the partner. If the participants were instructed that the partner was about to take their position in the next round, they could be more generous, hoping for the partner to repay the same amount. However, in Gurven’s (2004) study, the participants played a few economic games in a row. Perhaps, the more reciprocal nature of other games the participants played had influenced their decisions to share in DG. Further, in the original Gurven’s (2004) experiment, the participants were given 20 Bs by the experimenter, whereas, in our experiment this was 10 Bs. It means that the participants of Gurven’s (2004) experiment would keep on average 13.6 Bs, whereas our participants kept on average 9 Bs – in this way, the difference between the two studies seems less pronounced. Finally, as suggested by Gurven (2004) himself, “with an increasing reliance on market goods to reduce temporal variation in food- and health-related risks, households become more self-sufficient, and may be less likely to share”; thus, altruism may decrease with increasing market involvement. As our experiment was conducted 15 years after the original study by Gurven (2004), and during these years the Tsimane’ became more integrated to the local economy, the lower willingness to share might simply be a reflection of these changes. However, at the current stage of research it is hard to determine, which of these explanations are the most likely causes of the discrepancies in sharing patterns among the Tsimane’. A certain limitation of the present study is that we did not control the subjective value of presented goods. Although in both cultures the items were perceived as small gifts, it cannot be guaranteed that the applied items were perceived as equally valuable by the Tsimane and Poles. However, it should be noted that the main focus of the study were within-group comparisons.

To sum up, the results of our study indicate that in DG, generosity and willingness to share can be measured with various goods, such as food or small objects. These findings broaden the knowledge on methods used to study economic behaviors, and open new, different questions on bases of generosity and altruism.

## Author Contributions

Conceived and designed the experiments: PS, AN, AS; Performed the experiments: AN, MM, EV, TH; Analyzed and interpreted the data: PS, AO, AS; Wrote the manuscript: AO, PS, AS, MM, AN, TH.

## Conflict of Interest Statement

The authors declare that the research was conducted in the absence of any commercial or financial relationships that could be construed as a potential conflict of interest.
